# Comprehensive identification of *bHLH* transcription factors in *Litsea cubeba* reveals candidate gene involved in the monoterpene biosynthesis pathway

**DOI:** 10.3389/fpls.2022.1081335

**Published:** 2022-12-21

**Authors:** Jiahui Yang, Yicun Chen, Ming Gao, Liwen Wu, Shifa Xiong, Siqi Wang, Jing Gao, Yunxiao Zhao, Yangdong Wang

**Affiliations:** ^1^ State Key Laboratory of Tree Genetics and Breeding, Chinese Academy of Forestry, Beijing, China; ^2^ Research Institute of Subtropical Forestry, Chinese Academy of Forestry, HangZhou, Zhejiang, China

**Keywords:** bHLH gene family, plant secondary metabolism, *Litsea cubeba*, genome-wide identification, terpenoids

## Abstract

*Litsea cubeba* (Lour.) Person, an economically important aromatic plant producing essential oils, has lemon-like fragrance and 96.44–98.44% monoterpene contents. bHLH transcription factor plays an important role in plant secondary metabolism and terpene biosynthesis. In this study, we used bioinformatics to identify bHLH transcription factors in *L. cubeba*, 173 *bHLH* genes were identified from *L. cubeba* and divided these into 26 subfamilies based on phylogenetic analysis. The majority of bHLHs in each subfamily shared comparable structures and motifs. While *LcbHLHs* were unevenly distributed across 12 chromosomes, 10 tandem repeats were discovered. Expression profiles of bHLH genes in different tissues demonstrated that *LcbHLH78* is a potential candidate gene for regulating monoterpene biosynthesis. *LcbHLH78* and the terpene synthase *LcTPS42* showed comparable expression patterns in various tissues and fruit development stages of *L. cubeba*. Subcellular localization analysis revealed that LcbHLH78 protein localizes to the nucleus, consistent with a transcription factor function. Importantly, transient overexpression of *LcbHLH78* increased geraniol and linalol contents. Our research demonstrates that *LcbHLH78* enhances terpenoid biosynthesis. This finding will be beneficial for improving the quality of *L. cubeba* and provides helpful insights for further research into the control mechanism of *LcbHLH* genes over terpenoid biosynthesis.

## Introduction

*Litsea cubeba (Lour.)* Person, belonging to the Lauraceae family, as an important woody oil tree for a long time since its fruit is rich in essential oil (Zhao et al., 2020). Chemical studies show that the main volatile compounds in *L. cubeba* essential oil (LCEO) are monoterpenes, sesquiterpenes, and their derivatives (Chen et al., 2012). Meanwhile, LECO has become an essential component in the natural antibacterial industry because of the antibacterial and anti-inflammatory properties of terpenoids (Chen et al., 2020b). At present, ways to increase the content of geranial and neral, the main effective components of LCEO, is a hot topic in research on terpene metabolism (Chen et al., 2012). The biosynthetic pathway of monoterpenoids has been studied in many plants. Although terpenes have high structural diversity, they are derived from two isomeric basic backbone molecules, IPP and DMAPP, which are synthesized either through the MVA or MEP pathways (Sacchettini and Poulter, 1997; Sapir-Mir et al., 2008). The head-to-tail condensation of one DMAPP molecule with one IPP molecule forms geranyl diphosphate (GPP) (Wang and Ohnuma, 2000), then GPP synthases (GPPS) provide precursors for monoterpenes (Sun et al., 2016; Chen et al., 2019). Finally, monoterpenes parent scaffold is produced by monoterpene synthases (Chen et al., 2012). Although the key enzymes of the monoterpene biosynthesis pathway have been studied, improvement of LCEO quality based on the regulation of structural gene remains limited. Previous studies found that transcription factors (TFs) can coordinate the transcription of multiple metabolic pathways but also affect the transcription of genes in the same metabolic pathway (Rushton et al., 2010; Dubos et al., 2017; Zhang et al., 2017). Five TF families, including the basic helix-loop-helix (bHLH) family, are involved in the production of terpenoids in plants (Wang et al., 2021).

The bHLH TF family is one of the largest TF gene families in plants (Hong et al., 2012). For the bHLH domain, there are about 15 amino acids in its N-terminal region, and the primary function of these amino acids is to bind to cis-elements in the DNA (Murre et al., 1989). The C-terminal side of the bHLH domain, which comprises about 40 amino acids, aids in the formation of homo- and heterodimer complexes (Ferre-D’Amare et al., 1994). The bHLH family has been identified in a variety of plants thanks to the rapid advancement of genome sequencing technologies, for example, *Orchidaceae* (Zheng et al., 2021), *Prunus mume* (Wu et al., 2022), *Brassica oleracea* L. (Li et al., 2022), *Carthamus tinctorius* (Hong et al., 2019), *Aralia elata* (Wang, Y. et al., 2022), and others. Identification of bHLH transcription factors at a genome-wide level will enhance our understanding of the transcription and function of the *bHLH* gene family. At present, the regulation of plant terpenoids by bHLH TFs has been extensively reported, for example, the medicinal plant *Catharanthus roseus* (Van Moerkercke et al., 2015), *Phalaenopsis* (Chuang et al., 2018), *Betula platyphylla* (Yin et al., 2017), *Glycyrrhiza uralensi* (Tamura et al., 2018) and so on. Notably, members of the bHLH IIIe branch in *Arabidopsis* play a positive role in the regulation of plant secondary metabolism by jasmonic acid (JA) (Goossens et al., 2017).

Although the bHLH family has been identified to improve terpenoid production, the comprehensive identification of bHLH transcription factors in *L. cubeba* and the interpretation of their functions in regulating terpenoid biosynthesis pathway are still limited. In this study, we identified *bHLH* family genes in *L. cubeba* from transcriptome data and examined their functional annotations as well as the physicochemical characteristics, categorization, and conserved motif distribution of their proteins. In addition, we identified LcbHLHs that might be involved in terpene biosynthesis in *L. cubeba* by comparing their gene expression profiles with those of the terpene synthase *LcTPS42*. *LcbHLH78* was selected for functional study to verify its function in terpenoid biosynthesis. This study provides a theoretical basis for understanding the molecular mechanism underlying the regulation of terpenoid biosynthesis by *bHLH* TFs.

## Materials and methods

### Plant materials

The materials *L. cubeba* used in this study from HangZhou City, Zhejiang Province, China (30°27′94′′N, 119°58′ 43′′E). Collecting different tissues including root, stem, leaf, and flower of 5-year-old *L. cubeba*. The fruits of different developmental stages were collected at 10 a.m. on 30, 60, 90, 120, and 150 days after flowering, and immediately frozen in liquid nitrogen, then stored at -80°C for RNA extraction.

### Genome-wide identification of bHLH genes

The CDS sequences, protein sequences needed for analysis were obtained from *L. cubeba* genome database (Chen et al., 2020b). The *bHLH* gene sequence of *L. cubeba* was extracted using TBtools v1.0686 (https://github.com/CJ-Chen/TBtools), with the thresholds of the screening process set to 1e-5 and 45% filtration. Furthermore, putative *LcbHLH* proteins were discovered by reviewing HMMER and BLAST results and manually deleting duplicated sequences. Predicted *LcbHLH* genes were then double-checked using batches from the NCBICDD, SMART, and PFAM databases. Finally, 173 LcbHLH TFs were identified, and a phylogenetic tree was reconstructed using PhyML 3.0 with the default parameters (Gascuel, 2010).

### Sequence analyses of bHLH proteins and gene structure

LcbHLH protein sequences were uploaded to the ExPASy online program (Guo et al., 2014) to calculate their molecular weights (MW), isoelectric points (pI) and GRAVY values. To identify conserved motifs, the MEME (Bailey et al., 2015) 10 suite was applied using default settings. The gff3 file for the *L. cubeba* genome, which provides details of gene structure and was visualized using TBtools, was used to determine the exons and introns of each bHLH gene.

### Chromosomal location and collinearity of *bHLH* genes

BLAST programs were used to map *bHLH* gene sequences to *L. cubeba* chromosome survey sequences to determine the positions of *LcbHLH* genes on the 12 chromosomes. Precise gene-location results were displayed using MG2C V2.1software (http://mg2c.iask.in/mg2c_v2.1/). An interspecies collinearity analysis of *bHLH* genes was performed using MCscanX software.

### Cis-regulatory elements analysis of *TPS* genes

Promoter sequences of *L. cubeba* terpene synthase (TPS) family members were extracted using TBtools software and used to detect and visualize cis-acting elements. Detection and identification of cis-elements was carried out using PlantTFDB (http://planttfdb.gao-lab.org/) software.

### RNA extraction and quantitative reverse-transcription PCR

RNA of *L. cubeba* was extracted and reverse transcribed by the method provided by Zhao et al. (2020). qRT-PCR was carried out with the assistance of an ABI PRISM 7500 instrument and the TB Green^®^ Premix Ex TaqTM II kit. The actin gene from *L. cubeba* ubiquitin conjugating enzyme (UBC) was utilized as a reference gene (Chen et al., 2020). The total volume of the qRT-PCR system was 25 μL, which comprised the following components: 12.5 μL Green Premix Ex TaqII (Til RNaseH Plus) (2x) Mix, 1.0 μL upstream primer (10 μM), 1.0 μL downstream primer (10 μM), 2.0 μL cDNA, and 8.5 μL ddH2O. Reactions were prepared on ice. Each sample was prepared using three technical replicates in addition to a control that lacked cDNA. To calculate relative expression levels, the relative expression was calculated by 2^-ΔΔCT^ method. Findings are reported as the mean plus standard deviation across all three replicates. Primer Premier 3.0 was used to create primers for qRT-PCR reactions of the chosen *bHLH* genes, which are detailed in **Table S2**.

### Determination of subcellular localization

To make a prediction regarding the subcellular localization of *LcbHLH78* in *L. cubeba*, Euk-mPLoc 2.0 online software was utilized (Chou and Shen, 2010). Transient expression of *LcbHLH78* fusion protein in tobacco epidermal cells provided conclusive evidence for this assertion. *LcbHLH78* was cloned into the transient expression vector pNC-Green-SubC to generate a 35S:GFP-LcbHLH78 recombinant vector (Yan et al., 2021). Then, 35S:GFP-LcbHLH78 and the empty vector were transferred into *Agrobacterium* strain GV3101 by chemical conversion method (Wydro et al., 2006). The OD600 of the Agrobacterium suspension was adjusted to 0.8 using an infection solution containing 10 mM MES, 10 mM MgCl_2_, and 200 mM acetosyringone at pH 5.7 and incubated for 4 h at 28°C before infiltration into *Nicotiana Benthamiana* leaves that were 4-week-old. Fluorescence signals were examined using a confocal laser scanning microscope between 40 and 52 h after infiltration (ZEISS LSM 880, Germany). OsRde nuclear localization protein with red fluorescence was used as a positive control.

### Transient overexpression of *LcbHLH78* in *L. cubeba*

*LcbHLH78* transient overexpression analysis was performed using sterile seedlings of *L. cubeba*. For the preparation of sterile seedlings, cut about 6 mm with buds and stems and insert them into MS (Murashige and Skoog) basal medium containing, after 30 days of light culture, transfer to the new MS basal medium containing and continue to be cultured under light for 30-45 days. Sterile *L. cubeba* shoots were harvested and propagated in basal medium containing 6-BA, IBA, sugar, and agar (pH 5.8). After being individually transformed to *Agrobacterium* strain LBA4404, the empty vector (pNC-Cam2304-35S) and a recombinant vector containing *LcbHLH78* (pNC-Cam2304-35S-LcbHLH78) were infiltrated into leaves of sterile seedlings displaying similar growth and cultured at 26°C for 50 - 72 h. Then, collect the leaves with consistent growth state for qRT-PCR study and save the remaining leaf samples at –80°C for volatile analysis. Volatiles were examined using GC-MS method (Zhao et al., 2020). **Table S2** provides specific information on all primers.

## Results

### Identification and sequence analysis of *bHLH* genes

To identify *bHLH* TFs involved in terpenoid biosynthesis of LCEO, we first used an implicit Markov model to search for the bHLH domain using hmmsearch. We identified homology with *A. thaliana* bHLH proteins, and further screened candidate genes using NCBI-CDD conserved domain search. 173 bHLH proteins were filtered from the *L. cubeba* genome, named *LcbHLH1* - *LcbHLH173* according to their location on chromosomes or scaffolds. The molecular weight, hydrophilicity, and isoelectric point of each protein was calculated using the ExPASy tool, and the bHLH proteins range in size from 90 aa (*LcbHLH44*) to 1, 098 aa (*LcbHLH75*), theoretical isoelectric point ranged from 4.49 (*LcbHLH125*) to 11.51 (*LcbHLH126*), and hydropathicity values of all LcbHLH proteins varied between -0.083 and -1.062. More detailed information was shown in **Table S1**.

### Phylogenetic structure of LcbHLH proteins

**Figure 1:**
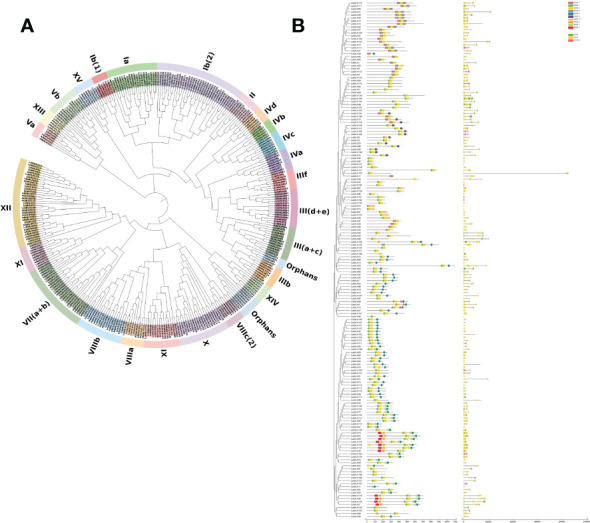
**(A)** Phylogenetic tree of bHLH proteins in *L. cubeba* and *A. thaliana*. Different colors represent different groups, and all *L. cubeba* bHLH proteins are clustered into subclades based on the priority classification rule of *A. thaliana* bHLH proteins. **(B)** Phylogenetic tree, gene structure, and conserved motif analyses of the *LcbHLH* family. Left: Multiple sequence alignment of bHLH domain sequences of *L. cubeba* performed using ClustalW. A neighbor-joining tree was reconstructed using MEGA X with 1,000 bootstrap replicates. Middle: Conserved motifs. MEME analysis revealed conserved motifs of LcbHLH proteins. Colored boxes on the right denote 10 motifs. Right: Gene structure. Yellow boxes, black lines, and green boxes represent exons, introns, and UTRs (untranslated regions), respectively.

To elucidate the structure and functions of *L. cubeba bHLH* TFs at the genomic level, we first reconstructed phylogenetic trees of *LcbHLH* and *AtbHLH* based on HMMER domain search and homology comparison with bHLH members in *A. thaliana*. The 173 *LcbHLHs* were classified into 26 groups according to the groups defined in *A. thaliana*; all subfamilies comprised members from both *L. cubeba* and *A. thaliana*, but the number of proteins differed between the two species (**Figure 1A**). The largest LcbHLH subgroups were XII and Ib (2), both with 35 members, while subgroup IVb was the smallest, with only five members: three in *A. thaliana* and two in *L. cubeba*. Twelve subgroups contained the same number of family members in *L. cubeba* and *A. thaliana*. Subgroup Ib (1) showed the greatest numerical discrepancy, with half as many members in *A. thaliana* as in *L. cubeba*. It is worth noting that seven bHLH family members clustered into the III (d+e) subfamily. Of these, *LcbHLH55*, *LcbHLH59*, *LcbHLH78*, and *LcbHLH123* belonged to the IIIe family. Proteins belonging to the subfamily IIIe are involved in the regulation of plant metabolism and induced by the defense-related hormone JA (Goossens et al., 2017).

### Conserved motif and structural analyses

To further study domains in LcbHLH, we analyzed the gene structure and conserved domains of 173 *L. cubeba* bHLH proteins. Two types of highly conserved protein motifs, denoted motif 1 and motif 2, were present in most sequences. Although there was considerable variation in the length of LcbHLHs amongst subfamilies, the lengths and positions of conserved motifs were similar, suggesting a phylogenetic relationship between them (**Figure 1B**). However, significant differences were found between the various subfamilies, and some motifs were only found in certain subfamilies. For example, motif 5 was only found in subfamilies III (d + e) and IIIf. This suggests that motif 5 may specifically function in these subfamilies.

We determined the exon-intron structure of *LcbHLH* genes based on their evolutionary classification. *LcbHLH* genes had between 0 and 12 introns, with 14 *LcbHLH* genes being intron-free, 15 *LcbHLH* genes having one intron, and the remaining genes having two or more introns. Furthermore, most *LcbHLH* genes belonging to the same subfamily had similar exon/intron distribution patterns. For instance, subfamily III (d + e) had 0 - 2 introns, while subfamily IIIf had 8 - 9 introns. Frequent occurrence of intron gains and losses during evolution can make gene structures more complex (Roy and Gilbert, 2005). However, exceptions were also found among these genes. For example, the members of subfamily Ib (2) had a differing number of introns and exhibited great diversity in exon length.

### Chromosomal arrangement and gene duplication of *LcbHLHs*

**Figure 2:**
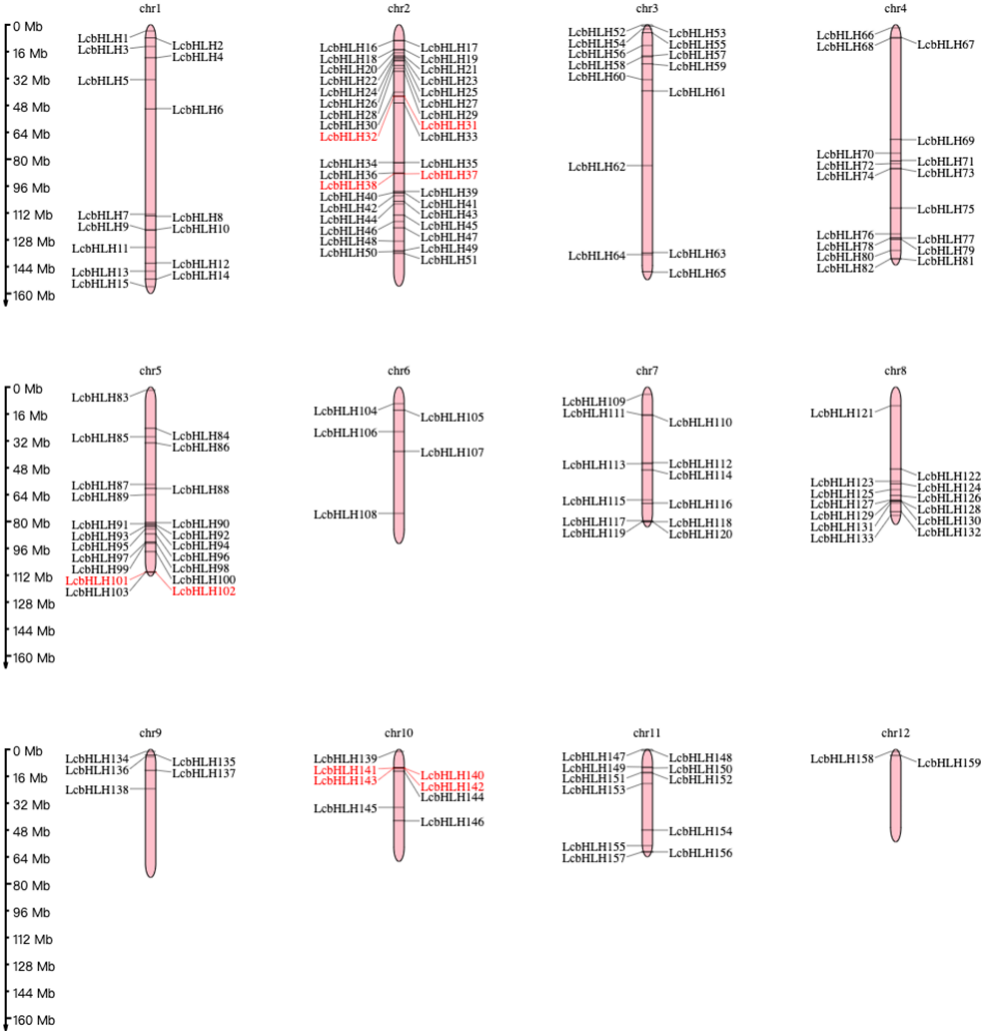
Chromosomal distribution of *LcbHLH* genes. *LcbHLH* genes (159) were unevenly mapped on 12 chromosomes. Tandem duplicated gene pairs are displayed with red color.

While 14 *LcbHLH* genes were localized on unassembled genomic scaffolds, 159 genes were localized unevenly on the 12 *L. cubeba* chromosomes. The most abundant chromosomal region was chromosome 2, harboring 36 *bHLH* genes, followed by Chr5 (21 genes), Chr4 (17 genes), Chr1 (15 genes), Chr3 (14 genes), Chr8 (13 genes), and Chr7 (12 genes); Chr12 had the fewest gene family members of any of the chromosomes, at 2 genes. We also discovered that the length of individual *L. cubeba* chromosomes varies. Chr1 has the longest arm, while Chr12 has the shortest length of any of the chromosomes. This demonstrated that there was no correlation between the distribution of *LcbHLH* genes and the length of chromosomes. Five pairs of *LcbHLH* genes mapping to chromosomes 2, 5, and 10 were characterized as tandem duplications among the 159 *LcbHLH* genes (**Figure 2**). IDs and genomic positions of the *LcbHLH* genes are listed in **Table S1**.

**Figure 3:**
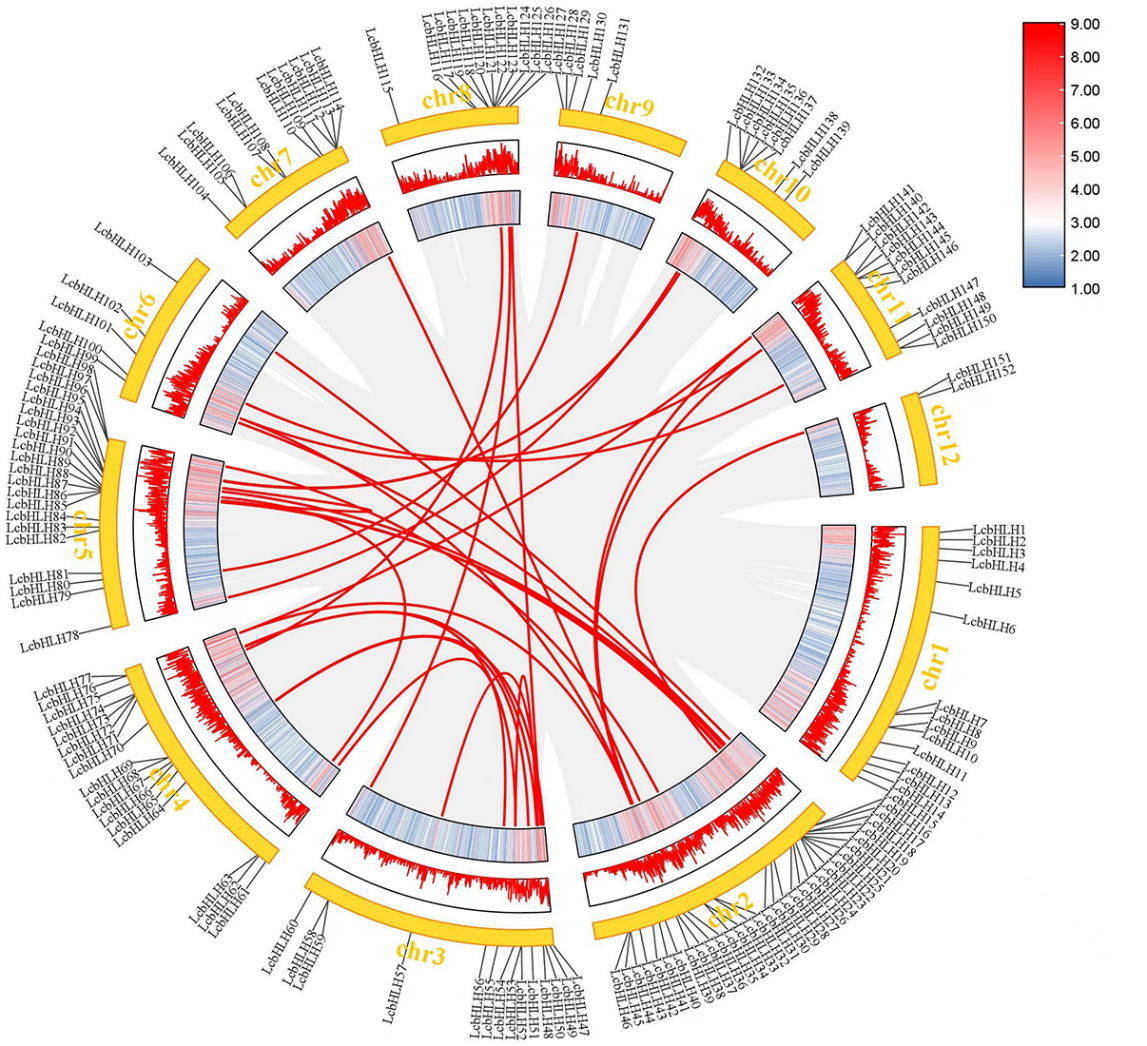
Collinearity analysis of *LcbHLH* genes. Circle plot created using the MCScanX tool. Collinear genes are linked by colored lines.

Genome duplication, tandem duplication, segmental duplication, and transposon duplication all contribute to the evolution of plants (Qiao et al., 2019). We created a syntenic map of *L. cubeba* to better understand the evolutionary process underlying the *LcbHLH* gene family. Many genes with collinearity were found on chromosomes 2, 3, and 5 (**Figure 3**). These findings suggest that gene duplication, particularly segmental duplication, may be linked to *LcbHLH* gene family amplification and that these duplication events may be the primary driver of *LcbHLH* evolution.

### Analysis of cis-acting regulatory elements of TPS

**Figure 4:**
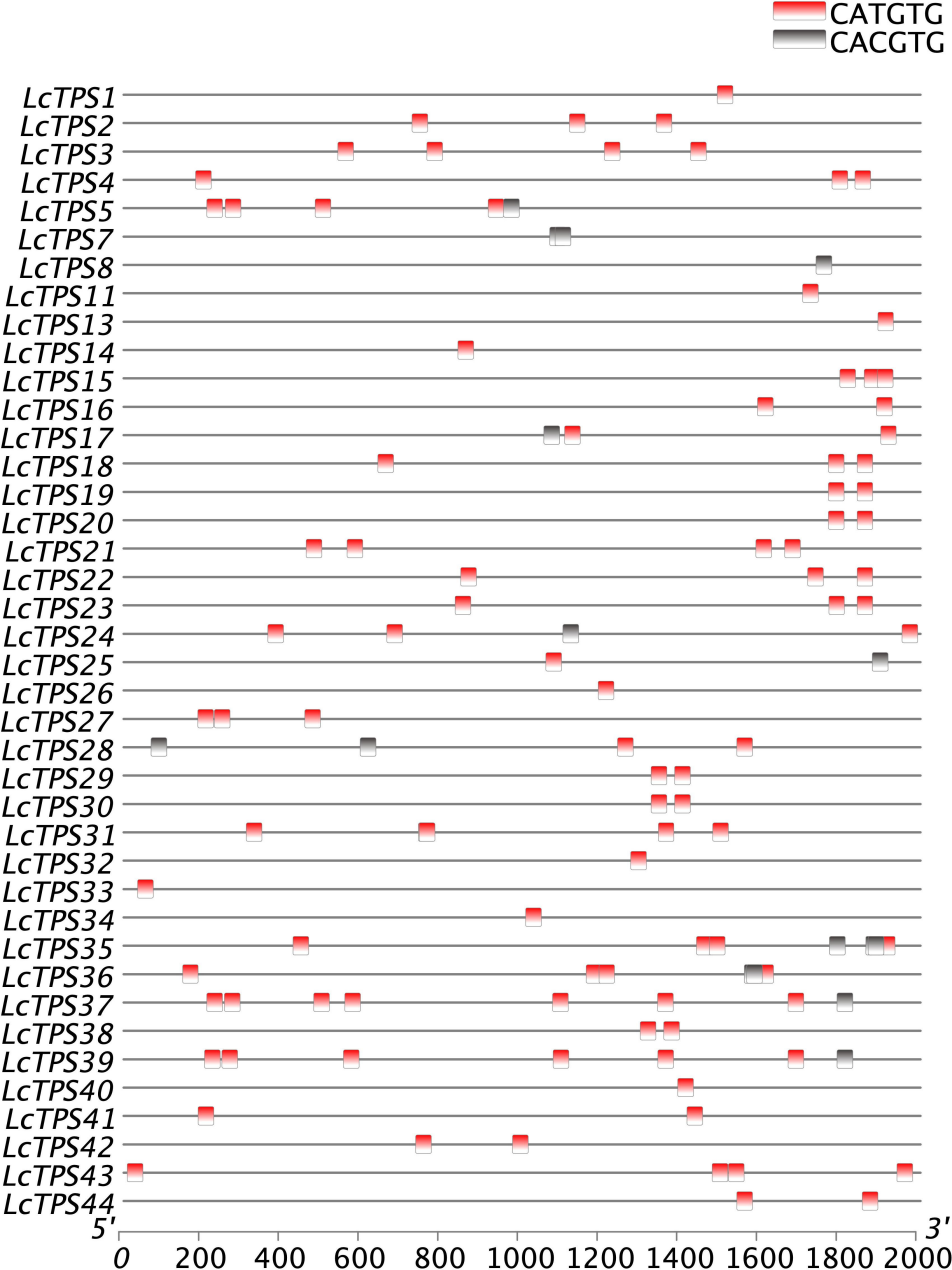
Cis-acting elements of *LcTPS* gene promoters. Promoter analysis was performed on 2000-bp sequences upstream of the transcription start sites.

We used PlantTDFB software to find and analyze probable cis-elements in the promoter regions of TPS genes, 2,000 bp upstream of the start codon, in order to further speculate the relationship between the *TPS* gene and bHLH transcription factors. The results show that the TPS promoter contains CACGTG/CATGTG sequences (**Figure 4**). In addition, previous research has shown that bHLH proteins regulate terpenoid biosynthesis by binding to G-box motifs found in the promoters of terpenoid biosynthesis genes (Liu et al., 2015; Wang et al., 2022). Considering the distribution of cis-elements in the promoter of these genes, we speculate that the bHLH TFs in *L. cubeba* also regulates terpenoid biosynthesis by binding to the G-box sequence on the TPS promoter.

### *LcbHLH78* and *LcTPS42* have similar expression patterns

**Figure 5:**
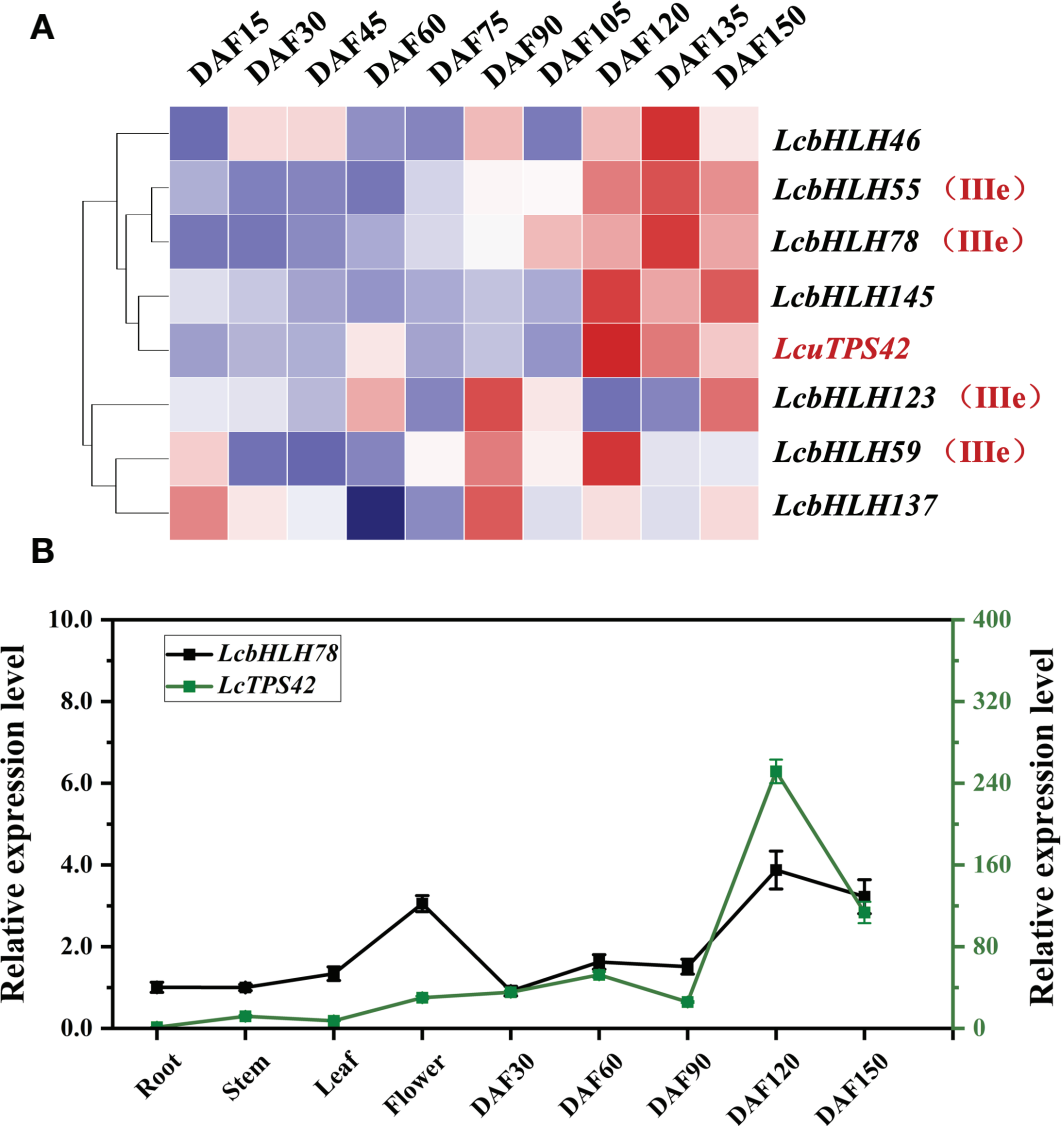
**(A)** Expression analysis of III (d+e) subfamily members and *LcTPS42* during pericarp development of *L. cubeba*. **(B)** Relative quantitative expression patterns of *LcbHLH78* and *LcTPS42* in *L. cubeba* fruit. DAF, days after full bloom. Data are means and standard deviations of three replicates. The *L. cubeba Ubiquitin conjugating enzyme* (*UBC*) gene served as a reference gene for internal control.

Monoterpenes are mainly produced in the *L. cubeba* pericarp. As a result, the *bHLH* TFs, which are abundant in the pericarp, are most likely to be candidate genes for regulating terpene synthesis. Additionally, *LcTPS42* has been identified as the key enzyme for geraniol, linalool and other monoterpenoids synthesis in the previous study, therefore, *LcTPS42* was also included as the reference for the co-expression analysis (Chen et al., 2020b). Transcriptome sequences (PRJNA763042) of *L. cubeba* pericarp at different stages of development were used to identify *LcbHLH* genes. The expression trends of several members of subgroup IIIe were consistent with that of *LcTPS42* in the pericarp of *L. cubeba* (**Figure 5A**). *LcbHLH78* was expressed throughout all developmental periods, with the highest expression at 120 days after full bloom highly consistent with the expression trend of *LcTPS42*, suggesting that *LcbHLH78* may be involved in the regulation of *LcTPS42* (**Figure 5B**).

### Subcellular localization of *LcbHLH78*

**Figure 6:**
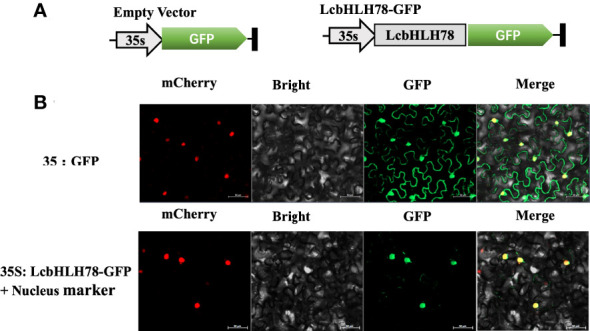
**(A)** Schematic diagram of vector; **(B)** *LcbHLH78* is localized in the nucleus. LcbHLH78-GFP and control plasmids were transiently expressed in *N. benthamiana* cells. Fluorescence was observed using a confocal fluorescence microscope at 36 h after incubation. Pictures show mCherry, bright, GFP, and Merge from left to right. Bar = 50 μm.

TFs typically carry out transcriptional regulatory tasks in the nucleus (Zhu et al., 2020). Therefore, we investigated the subcellular distribution of LcbHLH78 protein. GFP fluorescence of the empty vector was distributed throughout the cells of *N. benthamiana* leaves, while the nuclear marker and GFP-fused LcbHLH78 protein were localized in the nucleus (**Figure 6**).

### *LcbHLH78* promotes geraniol and linalool biosynthesis in *L. cubeba*

**Figure 7:**
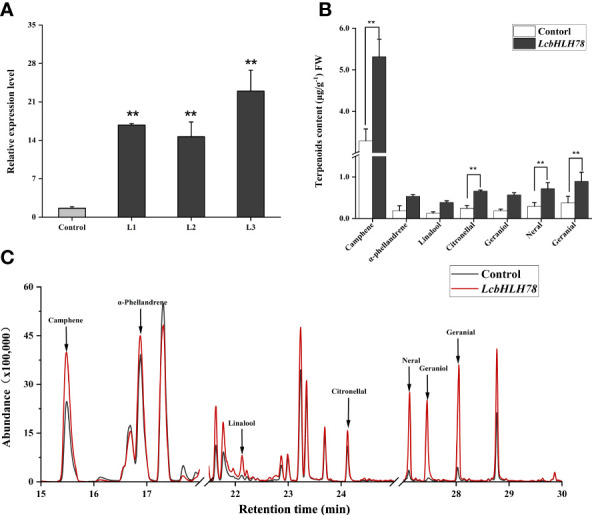
**(A)** Expression levels of *LcbHLH78* were significantly increased compared with the control (highlighted in red). **(B, C)** Terpenoid levels in sterile seedlings overexpressing *LcbHLH78* and sterile *L. cubeba* leaves infected with *Agrobacterium tumefaciens* containing *LcbHLH78* driven by the 35S promoter were determined using GC-MS. Data are shown as mean ± standard deviation of three replicates (**P < 0.01).

Because the stable transformation of *L. cubeba* is challenging, we used an efficient and simple transient expression assay to investigate the function of *LcbHLH78*. We transiently overexpressed *LcbHLH78* in *L. cubeba* following the transient transformation method of Wang M Y et al. (2022). After transient expression of *LcbHLH78*, we detected a 10-fold increase in *LcbHLH78* expression relative to that in seedlings transformed with an empty vector (**Figure 7A**). Transient overexpression of *LcbHLH78* enhanced the accumulation of α-phellandrene, linalool, citronellal, geraniol, neral, geranial, and camphene in *L. cubeba* leaves (**Figures 7B, C**). Previous studies revealed that *LcTPS42* is highly expressed in the pericarp and catalyzes the biosynthesis of geraniol and linalool (main components of monoterpenoid) in *L. cubeba* (Zhao et al., 2020). In this study, the contents of linalool, geraniol, and α-phellandrene were significantly increased after transient expression of *LcbHLH78*, consistent with *LcTPS42* catalyzing formation of monoterpene components. It is worth noting that geraniol is the direct precursor of citral, a key component of the essential oil in *L. cubeba* fruit, with linalool and α-pinene as the main monoterpene components. In addition, camphene contents were also significantly increased after transient expression of *LcbHLH78*. Actually, G-box elements were also found on the promoters of genes involved in MVA and MEP pathways. The overexpressing of *LcbHLH78* not only activated the expression of *LcTPS42*, but also the pathway (**Figure S1**). These findings suggest that *LcbHLH78* as a candidate gene enhances terpenoid biosynthesis.

## Discussion

### Gene duplication offers the evolution dyamic for expansion of the *Litsea cubeba bHLH* gene family

bHLH TFs, an important set of eukaryotic protein family members, play a critical function in the growth, development, and secondary metabolism of organisms (Zhao et al., 2018). Up to now, bHLH TFs have been identified in a variety of plants, such as *Dracaena cambodiana*, *Pyrus bretschneideri*, and *Persian walnut* (Zhu et al., 2020; Dong et al., 2021; Ullah et al., 2021). In our study, 173 *LcbHLH* genes were identified, and these were separated into 26 different subfamilies based on the phylogenetic relationships they shared with other *bHLH* genes found in *Arabidopsis* (Li et al., 2006). Among them, subgroup IIIe, which is responsible for the regulation of secondary metabolism, contains four members (*LcbHLH55*, *LcbHLH59*, *LcbHLH78*, *LcbHLH123*). In terms of the number of genes, there are more *bHLH* gene family members in *L. cubeba* than in some other species: 173 *LcbHLHs* compared with 162 *ArbHLH* genes (Bailey et al., 2003) and 152 *SlybHLH* genes (Wang et al., 2015). This may be because two whole-genome replication (WGD) events occurred in the *L. cubeba* genome (Chen et al., 2020b). In addition, other plants have a larger number of *bHLH* gene family members than *L. cubeba*; for example, wheat has 225 *bHLH* genes (Guo and Wang, 2017), tobacco has 190 *bHLH* genes (Rushton et al., 2008), and sorghum has 174 *bHLH* genes (Fan et al., 2021).

Tandem duplication and segmental duplications provide different evolutionary dynamics to duplicated genes (Leister, 2004; Panchy et al., 2016). *Arabidopsis* and rice genomes contain about 10% tandem repeat genes, which have made important contributions to the expansion of some large gene families (Blanc et al., 2000). The evolutionary history of *LcbHLH* genes shows that tandem duplications and segmental duplications have contributed to the expansion of *LcbHLH* genes. We speculate that 10 *LcbHLH* genes are associated with tandem repeat events, and large-fragment replication events involve different chromosomes (McGowan et al., 2020). These duplicated *LcbHLH* genes probably formed new gene functions to adapt to various growth conditions. Repetitive genes play an important role in plant adaptation to complex and changeable environments (Cannon et al., 2004; Wang et al., 2022). Short-term and long-term evolutionary retention mechanisms of repetitive genes include subfunctionalization, new functionalization, and loss (Chen et al., 2019). After gene replication, sequence differentiation of two homologous gene copies in the promoter region may lead to expression differentiation between them. Previous studies have shown that two genes adjacent to each other on the chromosome are more likely to be co-regulated, especially two tandem repeat genes (Wang et al., 2022). In this study, sequence alignment of gene family members in the IIIe branch of *L. cubeba* revealed high levels of similarity. Some amino acids had undergone mutation, however, which may be the cause of functional differentiation.

### *Litsea cubeba bHLH* genes may play important roles in terpenoid biosynthesis

*bHLH* TF is one of the largest families of transcription factors in plants, which participates in the regulation of plant growth and development, signal transduction, and responding to abiotic stresses such as drought, low temperature, salt and heavy metals in plants (Goossens et al., 2017). Meanwhile, *bHLH* plays an important role in secondary metabolism, especially terpenoid biosynthesis. For example, transcription factor BpbHLH9 in birch can increase the content of triterpenoids and the expression of key genes (Yin et al., 2017); two tissue-specific bHLH transcription factors, BI and BT, are involved in gene expression regulation of triterpenoid synthesis, which has important contributions to the cultivation and selection of cucumber (Shang et al., 2014).

Terpenoids are one of the most significant pharmacologically active components of *L. cubeba*, and the amount of these compounds directly influences the economic value of this plant. However, biosynthesis of the secondary metabolite terpene is tissue-specific and spatiotemporal. As a result, tissue-specific expression of TF genes may have a significant influence on the production of terpenes. We found that many *LcbHLH* genes are constitutively expressed at various stages of fruits. The expression trend of several subgroup IIIe members was consistent with that of *LcTPS42*, which was highly expressed in the later phases of fruit peel growth (**Figure 6**). Interestingly, essential oil is rapidly produced during the later phases of fruit peel growth in *L. cubeba*. We speculate that the differential expression of LcbHLH family members in different tissues leads to their different secondary metabolite contents (Wang et al., 2021). Tissue-specific expression has also been extensively studied in a variety of plants. During the development of *Ficus carica L*. fruit, members of distinct bHLH subfamilies are expressed differently in the female flower tissue and peel (Song et al., 2021). Four genes involved in anthocyanin biosynthesis in walnut show similar expression patterns in the leaf and peel of red and green walnut at different developmental stages (Zhao et al., 2021). *bHLH* genes are mostly expressed in the leaves and stems of *Artemisia argyi*, with reduced expression in roots (Yi et al., 2022). All these studies indicate that *bHLH* genes belonging to the same subfamily have similar and tissue-specific expression patterns.

### *LcbHLH78* play a positive role in terpenoid biosynthesis by regulating *LcTPS42*

*bHLH* TFs can regulate the production of terpenoids by directly binding to the promoter of key genes involved in the biosynthesis pathway. Such as *AtMYC2* activates *TPS21* and *TPS11* synthase genes to increase the release of sesquiterpenes by binding to the promoter region of these genes in *A. thaliana* (Hong et al., 2012). SlJIG, a bHLH TF, was found to be directly downstream of MYC2, regulating the expression of *TPS* genes or participating in the classical JA defense pathway, and is predicted to participate in JA-induced terpenoid biosynthesis (Cao et al., 2022). In *Freesia hybrida*, genes encoding three TFs, *FhMYB21L1*, *FhMYB21L2*, and *FhMYC2*, were isolated and functionally verified as regulators of linalool biosynthesis (Yang et al., 2020). Taken together, these studies suggest that.

According to previous research, the terpenoids in *L. cubeba* were mainly accumulated in the pericarp. The expression level of the fruit varies with different developmental stages, and the highest expression level is generally found between 120d and 150d after flowering (Wang et al., 2022). We found the expression patterns of the *bHLH* gene and *LcTPS42* in different tissues and developmental stages in the heat map, *LcbHLH46*, *LcbHLH55*, *LcbHLH78*, *LcbHLH145* are co-expressed with *LcTPS42*, however, we choose *LcbHLH78* as terpene synthesis candidate gene, mainly because *LcbHLH55* has been studied before (Wang et al., 2022), while *LcbHLH46* and *LcbHLH145* do not belong to IIIe branch, which may not play a positive role in JA regulation of plant secondary metabolism. Furthermore, Terpenoid biosynthesis can also be promoted by exogenous MeJA (Ji et al., 2019; Wang et al., 2022). In our study, GC-MS was used to determine the main terpenoid in *L. cubeba*, impressively, overexpression of *LcbHLH78* increased the α-pinene, linalool, geraniol, neral, and geranial. Thereinto, geraniol was the direct precursor of citral, a main component of the LECO, with linalool and α-pinene as the main monoterpene component (Chen et al., 2020). Different from *LcbHLH55*, GCMS volatiles that overexpressed *LcbHLH78* showed significantly increased camphene content, which may be due to the different functions of different transcription factors in terpene biosynthesis. This study provides a theoretical basis for the regulation of LCEO.

## Conclusion

In this study, we identification of the *LcbHLH* gene family, and a particular focus on candidate *LcbHLH78* gene function in *L. cubeba* terpenoid biosynthesis. The gene structure, chromosomal distribution, gene duplication, as well as the interactions and subcellular localization of LcbHLH proteins were analyzed. We functionally identified that *LcbHLH78*, a member of the IIIe bHLH TFs, controls geraniol and linalool biosynthesis. Furthermore, the expression profile of *LcbHLH78* in the pericarp is similar to that of *LcTPS42*. Our results indicate that *LcbHLH78* promotes geraniol and linalool biosynthesis, likely through the activation of *LcTPS42* expression. Altogether, this study lays the foundation for elucidating the biological and molecular functions of *L. cubeba* bHLH TFs.

## Data availability statement

The original contributions presented in the study are publicly available. This data can be found here: NCBI, PRJNA763042.

## Author contributions

YW, YZ, and JY carried out the molecular studies, participated in the analysis and drafted the manuscript; MG, LW provided technical and materials for experiments assistance; SW, JG provide revision of the spelling and format of the full text of this paper; YW, YZ and YC conceived the project, supervised the analysis, and critically complemented the manuscript. All authors contributed to the article and approved the submitted version.

## Supplementary material

Supplementary Figure 1 Cis-element analysis of terpenoids synthesis pathway gene promoters in *L. cubeba*. The potential cis-regulatory elements in the promoter regions 2,000 bp upstream of the *L. cubeba*. were predicted by PlantTDFB software.

## References

[B1] Bailey T. L. Johnson J. Grant C. E. Noble W. S. (2015). The MEME suite. Nucleic Acids Res. 43, W39–W49. doi: 10.1093/nar/gkv416 25953851 PMC4489269

[B2] Bailey P. C. Martin C. Toledo-Ortiz G. Quail P. H. Huq E. Heim M. A.. (2003). Update on the basic helix-loop-helix transcription factor gene family in arabidopsis thaliana. Plant Cell. 15, 2497–2501. doi: 10.1105/tpc.151140 14600211 PMC540267

[B3] Blanc G. Barakat A. Guyot R. Cooke R. Delseny M. (2000). Extensive duplication and reshuffling in the arabidopsis genome. Plant Cell. 12, 1093–1101. doi: 10.1105/tpc.12.7.1093 10899976 PMC149051

[B4] Cannon S. B. Mitra A. Baumgarten A. Young N D. May G. (2004). The roles of segmental and tandem gene duplication in the evolution of large gene families in arabidopsis thaliana. BMC Plant Biol. 4, 10. doi: 10.1186/1471-2229-4-10 15171794 PMC446195

[B5] Cao Y. Liu L. Ma K. Wang W. Lv H. Wang X.. (2022). The jasmonate-induced bHLH gene SlJIG functions in terpene biosynthesis and resistance to insects and fungus. J. Integr. Plant Biol. 64 (5), 14. doi: 10.1111/jipb.13248 35293128

[B6] Chen C. J. Chen H. Zhang Y. Thomas H. R. Frank M. H. He Y. H.. (2020). TBtools: An integrative toolkit developed for interactive analyses of big biological data. Mol. Plant 13, 1194–1202. doi: 10.1016/j.molp.2020.06.009 32585190

[B7] Chen C. J. Tseng Y H. Chu F H. Wei T Y. Cheng W W. Chen Y T.. (2012). Neuropharmacological activities of fruit essential oil from litsea cubeba persoon. J. Wood SC. 58 (6), 538–543. doi: 10.1007/s10086-012-1277-3

[B8] Chen H. Kllner T. G. Li G. Wei G. Chen F. (2019). Combinatorial evolution of a terpene synthase gene cluster explains terpene variations in oryza. Plant Physiol. 182 (1), 00948.2019. doi: 10.1104/pp.19.00948 PMC6945850 31712306

[B9] Chen Y. C. Li Z. Zhao Y. X. Gao M. Wang J. Liu K.. (2020b). The litsea genome and the evolution of the laurel family. Nat. Commun. 11 (1), 1675. doi: 10.1038/s41467-020-15493-5 32245969 PMC7125107

[B10] Chen X. Wang D. D. Fang X. Chen X. Y. Mao Y. B. (2019). Plant specialized metabolism regulated by jasmonate signaling. Plant Cell Physiol. 60 (12), 2638–2647. doi: 10.1093/pcp/pcz161 31418777

[B11] Chou K. C. Shen H. B. (2010). A new method for predicting the subcellular localization of eukaryotic proteins with both single and multiple sites: Euk-mPLoc 2.0. PLos One 5, e9931. doi: 10.1371/journal.pone.0009931 20368981 PMC2848569

[B12] Chuang Y. C. Hung Y. C. Tsai W. C. Tsai W. C. Chen W.H. Chen H. H. (2018). PbbHLH4 reg- ulates floral monoterpene biosynthesis in phalaenopsis orchids[J]. J. Exp. Bot. 69 (18), 4363–4377. doi: 10.1093/jxb/ery246 29982590 PMC6093345

[B13] Dong H. Chen Q. Dai Y. Hu W. Huang X. (2021). Genome-wide identification of PBrbHLH family genes, and expression analysis in response to drought and cold stresses in pear (pyrus bretschneideri). BMC Plant Biol. 21 (1), 86. doi: 10.1186/s12870-021-02862-5 33563216 PMC7874673

[B14] Dubos C. Stracke R. Grotewold E. Weisshaar B Martin C. Lepiniec L.. (2017). MYB transcription factors in arabidopsis. Trends Plant Sci. 15, 573–581. doi: 10.1016/j.tplants.2010.06.005 20674465

[B15] Fan Y. Yang H. Lai D. L. He A. L. Xue G. X. Feng L.. (2021). Genome-wide identification and expression analysis of the bHLH transcription factor family and its response to abiotic stress in sorghum [Sorghum bicolor (L.) moench]. BMC Genomics 22, 9. doi: 10.1186/s12864-021-07652-9 34090335 PMC8178921

[B16] Ferre-D’Amare A. R. Pognonec P. Roeder R. G. Burley S. K. (1994). Structure and function of the b/HLH/Z domain of USF. EMBO J. 13, 180–189. doi: 10.1002/j.1460-2075.1994.tb06247.x 8306960 PMC394791

[B17] Gascuel O. (2010). New algorithms and methods to estimate maximum-likelihood phylogenies: Assessing the performance of PhyML 3.0. Systematic Biol. 59 (3), 307–321. doi: 10.1093/sysbio/syq010 20525638

[B18] Goossens J. Mertens J. Goossens A. (2017). Role and functioning of bHLH transcription factors in jasmonate signalling. J. Exp. Bot. 68 (6), 1333–1347. doi: 10.1093/jxb/erw440 27927998

[B19] Guo C. Guo R. Xu X. Gao M. Li X. Song J.. (2014). Evolution and expression analysis of the grape (Vitis vinifera l.) WRKY gene family. J. Exp. Bot. 65, 1513–1528. doi: 10.1093/jxb/eru007 24510937 PMC3967086

[B20] Guo X. Wang J. (2017). Global identification, structural analysis and expression characterization of bHLH transcription factors in wheat. BMC Plant Bio. 17, 90. doi: 10.1186/s12870-017-1038-y 28558686 PMC5450219

[B21] Hong Y. Q. Ahmad N. Tian Y. Y. Liu J. Y. Wang L. Y. Wang G.. (2019). Genome-wide identification, expression analysis, and subcellular localization of carthamus tinctorius bHLH transcription factors. Int. J. Mol. Sci. 20, 3044. doi: 10.3390/ijms20123044 31234449 PMC6627405

[B22] Hong G. J. Xue X. Y. Mao Y. B. Wang L. J. Chen X. Y. (2012). Arabidopsis MYC2 interacts with DELLA proteins in regulating sesquiterpene synthase gene expression. Plant Cell. 24 (6), 2635–2648. doi: 10.1105/tpc.112.098749 22669881 PMC3406894

[B23] Ji J. J. Feng Q. Sun H. F. Zhang X. J. Li X. X. Li J. K.. (2019). Response of bioactive metabolite and biosynthesis related genes to methyl jasmonate elicitation in codonopsis pilosula. Molecules 24, 24030533. doi: 10.3390/molecules24030533 PMC6385095 30717158

[B24] Leister D. (2004). Tandem and segmental gene duplication and recombination in the evolution of plant disease resistance gene. Trends Genet. Tig 20 (3), 116–122. doi: 10.1016/j.tig.2004.01.007 15049302

[B25] Li X. Duan X. Jiang H. Sun Y. Tang Y. Yuan Z.. (2006). Genome-wide analysis of basic/helix-loop-helix transcription factor family in rice and arabidopsis. Plant Physiol. 141, 1167–1184. doi: 10.1104/pp.106.080580 16896230 PMC1533929

[B26] Li L. S. Zhang H. Chai X.H. Lv J. Hu L. L. Wang J.. (2022). Genome-wide identification and expression analysis of the MYC transcription factor family and its response to sulfur stress in cabbage (Brassica oleracea l.). Gene., 814, 146116. doi: 10.1016/j.gene.2021.146116 34942321

[B27] Liu Y. Ji X. Nie X. Qu M. Zheng L. Tan Z.. (2015). Arabidopsis AtbHLH112 regulates the expression of genes involved in abiotic stress tolerance by binding to their e-box and GCG-box motifs. New Phytol. 207, 692–709. doi: 10.1111/nph.13387 25827016

[B28] McGowan J. O'Hanlon R. Owens R. A. Fitzpatrick D. A. (2020). Comparative genomic and proteomic analyses of three widespread phytophthora species: Phytophthora chlamydospora, phytophthora gonapodyides and phytophthora pseudosyringae. Microorganisms. 8, 653. doi: 10.3390/microorganisms8050653 32365808 PMC7285336

[B29] Murre C. McCaw P. S. Baltimore D. (1989). A new DNA binding and dimerization motif in immunoglobulin enhancer binding, daughterless, MyoD, and myc proteins. Cell. 56, 777–783. doi: 10.1016/0092-8674(89)90682-x 2493990

[B30] Panchy N. Lehti-Shiu M. Shiu S. H. (2016). Evolution of gene duplication in plants. Plant Physiol. 171, 2294–2316. doi: 10.1104/pp.16.00523 27288366 PMC4972278

[B31] Qiao X. Li Q. Yin H. Qi K. Li L. Wang R.. (2019). Gene duplication and evolution in recurring polyploidization-diploidization cycles in plants. Genome Biol. 20, 38. doi: 10.1186/s13059-019-1650-2 30791939 PMC6383267

[B32] Roy S. W. Gilbert W. (2005). Rates of intron loss and gain: implications for early eukaryotic evolution. Proc. Natl. Acad. Sci. U.S.A. 102, 5773–5778. doi: 10.1073/pnas.0500383102 15827119 PMC556292

[B33] Rushton P. J. Bokowiec M. T. Han S. C. Zhang H. B. Brannock J. F. Chen X. F. (2008). Tobacco transcription factors: novel insights into transcriptional regulation in the solanaceae. Plant Physiol. 147, 280–295. doi: 10.1104/pp.107.114041 18337489 PMC2330323

[B34] Rushton P. J. Somssich I. E. Ringler P. Shen Q. J. (2010). WRKY transcription factors. Trends Plant Sci. 15, 247–258. doi: 10.1016/j.tplants.2010.02.006 20304701

[B35] Sacchettini J. C. Poulter C. D. (1997). Creating isoprenoid diversity. Science 277, 1788–1789. doi: 10.1126/science.277.5333.1788 9324768

[B36] Sapir-Mir M. Mett A. Belausov E. Tal-Meshulam S. Frydman A. Gidoni D.. (2008). Peroxisomal localization of arabidopsis isopentenyl diphosphate isomerases suggests that part of the plant isoprenoid mevalonic acid pathway is compartmen- talized to peroxisomes. Plant Physiol. 148 (3), 1219–1228. doi: 10.1104/pp.108.127951 18988695 PMC2577245

[B37] Shang Y. Ma Y. Zhou Y.. (2014). Biosynthesis, regulation, and domestication of bitterness in cucumber. Science 346 (6213), 1084–1088. doi: 10.1126/science.1259215 25430763

[B38] Song M. Wang H. Wang Z. Huang H. Chen S and Ma H. (2021). Genome-wide characterization and analysis of bHLH transcription factors related to anthocyanin biosynthesis in fig (Ficus carica l.). Front. Plant Sci. 12. doi: 10.3389/fpls.2021.730692 PMC8531510 34691109

[B39] Sun P. Schuurink R. C. Caissard J. C. Hugueney P. Baudino S. (2016). Myway: non- canonical biosynthesis pathways for plant volatiles. Trends Plant Sci 21 (10), 884–894. doi: 10.1016/j.tplants.2016.07.007 27475252

[B40] Tamura K. Yoshida K. Hiraoka Y. Sakaguchi D. Chikugo A. Mochida K.. (2018). The basic helix-Loop-Helix transcription factor GubHLH3 positively regulates soyasaponin biosynthetic genes in glycyrrhiza uralensis. Plant Cell Physiol. 59 (4), 778–796. doi: 10.1093/pcp/pcy046 29648666

[B41] Ullah I. Hou N. Yan F. Khan H. Zhao P. (2021). JrbHLH gene family: Genome-wide identification and transcriptional expression in Persian walnut (Juglans regia l.). Polish J. Environ. Stud. 30 (2), 1831–1839. doi: 10.15244/pjoes/123113

[B42] Van Moerkercke A. Steensma P. Schweizer F. Pollier J. Gariboldi I. Payne R.. (2015). The bHLH transcription factor BIS1 controls the iridoid branch of the monoterpenoid indole alkaloid pathway in catharanthus roseus. Proc. Natl. Acad. Sci. U.S.A. 112 (26), 8130–8135. doi: 10.1073/pnas.1504951112 26080427 PMC4491741

[B43] Wang M. Y. Gao M. Zhao Y. X. Chen Y. Y. Wu L. W. Yin H. F.. (2022). LcERF19, an AP2/ERF transcription factor from litsea cubeba, positively regulates geranial and neral biosynthesis. Horticult. Res. 9, uhac093. doi: 10.1093/hr/uhac093 PMC9327096 35912071

[B44] Wang J. Hu Z. Zhao T. Yang Y. Chen T. Yang M.. (2015). Genome-wide analysis of bHLH transcription factor and involvement in the infection by yellow leaf curl virus in tomato (Solanum lycopersicum). BMC Genomics 16, 39. doi: 10.1186/s12864-015-1249-2 25652024 PMC4333901

[B45] Wang M. Y. Jiao Y. L. Zhao Y. X. Gao M. Wu L. W. Wang S. Q.. (2022). Phytohormone and transcriptome of pericarp reveals jasmonate and LcMYC2 are involved in neral and geranial biosynthesis in litsea cubeba. Ind. Crops Products. 177, 114423. doi: 10.1016/j.indcrop.2021.114423

[B46] Wang K. C. Ohnuma S. I. (2000). Isoprenyl diphosphate synthases. Biochim. Biophys. Acta 1529 (1), 33–48. doi: 10.1016/s1388-1981(00)00136-0 11111076

[B47] Wang M. Z. Qiu X. X. Pan X. Li C. L. (2021). Transcriptional factor-mediated regulation of active component biosynthesis in medicinal plants. Curr. Pharm. Biotechnol 22 (6), 848–866. doi: 10.2174/1389201021666200622121809 32568019

[B48] Wang Y. Zhang H. Ri H. C. An Z. Y. Wang X. Zhou J. N.. (2022). Deletion and tandem duplications of biosynthetic genes drive the diversity of triterpenoids in aralia elata. Nat. Commun. 13, 2224. doi: 10.1038/s41467-022-29908-y 35468919 PMC9038795

[B49] Wu Y. Y. Wu S. H. Wang X. Q. Mao T. Y. Bao M. Z. Zhang J. W.. (2022). Genome-wide identification and characterization of the bHLH gene family in an ornamental woody plant prunus mume. Hortic. Plant J 16 (1), 9. doi: 10.1186/s12864-014-1209-2

[B50] Wydro M. Kozubek E. Lehmann P. (2006). Optimization of transient agrobacterium-mediated gene expression system in leaves of nicotiana benthamiana. Acta Biochim. Polonica. 53 (2), 289–298. doi: 10.18388/abp.2006_3341 16582986

[B51] Yang Z. Li Y. Gao F.. (2020). MYB21 interacts with MYC2 to control the expression of terpene synthase genes in flowers of freesia hybrida and arabidopsis thaliana. J. Exp. Bot 71 (14), 4140–4158. doi: 10.1093/jxb/eraa184 32275056

[B52] Yan Y. Wang P. Lu Y. Bai Y. Wei Y. Liu G.. (2021). MeRAV5 promotes drought stress resistance in cassava by modulating hydrogen peroxide and lignin accumulation. Plant J. 107, 847–860. doi: 10.1111/tpj.15350 34022096

[B53] Yin J. Li X. Zhan Y. Li Y. Qu Z. Sun L.. (2017). Cloning and expression of bp⁃ MYC4 and BpbHLH9 genes and the role of BpbHLH9 in triterpenoid synthesis in birch[J]. BMC Plant Biol. 17 (1), 214. doi: 10.1186/s12870-017-1150-z 29162040 PMC5698961

[B54] Yi X. Wang X. Wu L. Wang M. Yang L. Liu X.. (2022). Integrated analysis of basic helix loop helix transcription factor family and targeted terpenoids reveals candidate AarbHLH genes involved in terpenoid biosynthesis in artemisia argyi. Front. Plant Sci. 12. doi: 10.3389/fpls.2021.811166 PMC8801783 35111184

[B55] Zhang F. Fu X. Lv Z. Lu X. Shen Q. Zhang L.. (2017). A basic leucine zipper transcription factor, AabZIP1, connects abscisic acid signaling with artemisinin biosynthesis in artemisia annua. Mol. Plant 8, 163–175. doi: 10.1016/j.molp.2014.12.004 25578280

[B56] Zhao Y. Chen Y. Gao M. Yin H. Wu L. Wang Y.. (2020). Overexpression of geranyl diphosphate synthase small subunit 1 (LcGPPS.SSU1) enhances the monoterpene content and biomass. Ind. Crops Products. 143, 111926. doi: 10.1016/j.indcrop.2019.111926

[B57] Zhao F. Li G. Hu P. Zhao X. Li L. Wei W.. (2018). Identification of basic/helix-loop-helix transcription factors reveals candidate genes involved in an- thocyanin biosynthesis from the strawberry white-flesh mutant. Sci. Rep. 8, 2721. doi: 10.1038/s41598-018-21136-z 29426907 PMC5807450

[B58] Zhao W. Liu Y. Li L. Meng H. Yang Y. Dong Z.. (2021). Genome-wide identification and characterization of bHLH transcription factors related to anthocyanin biosynthesis in red walnut (Juglans regia l.). Front. Genet. 12. doi: 10.3389/fgene.2021.632509 PMC7943622 33719341

[B59] Zheng Q. D. Wang Y. OU Y. Ke Y. J. Yao Y. H. Wang M. J.. (2021). Research advances of genes responsible for flower colors in orchidaceae. Acta Hortic. Sinica. 48, 2057–2072. doi: 10.16420/j.issn.0513-353x.2021-0444

[B60] Zhu J. H. Xia D. N. Xu J. Guo D. Peng S. Q. (2020). Identification of the bHLH gene family in dracaena cambodiana reveals candidate genes involved in flavonoid biosynthesis. Ind. Crops Products. 150, 112407. doi: 10.1016/j.indcrop.2020.112407

