# Supplementary figures and images for: Comprehensive identification of bHLH transcription factors in Litsea cubeba reveals candidate gene involved in the monoterpene biosynthesis pathway
Source: Front Plant Sci. 2022 Dec 21;13:1081335. doi: 10.3389/fpls.2022.1081335 (PMC9811127; doi:10.3389/fpls.2022.1081335)

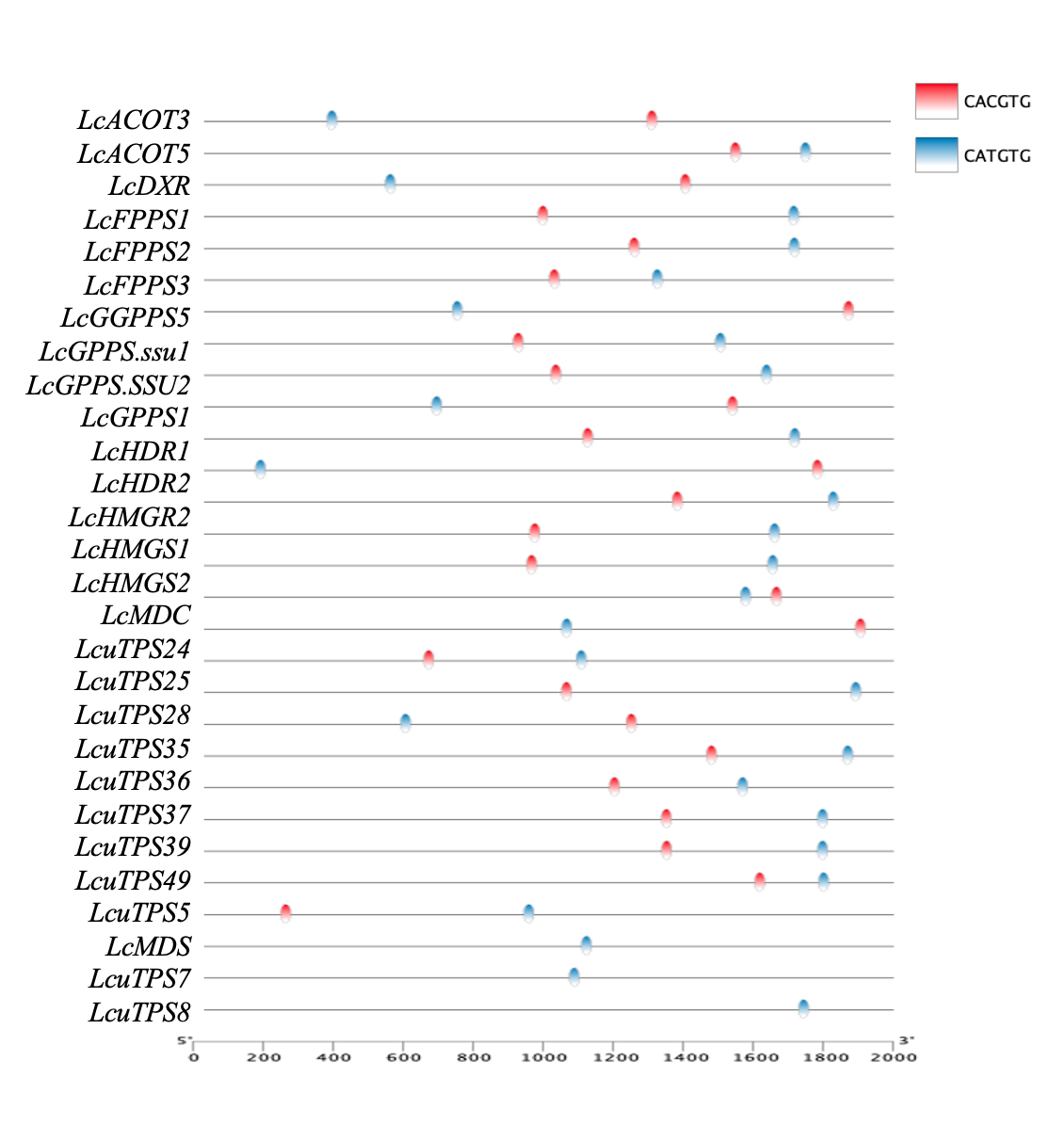

Supplement: Supplementary Figure 1 — Cis-element analysis of terpenoids synthesis pathway gene promoters in L. cubeba. The potential cis-regulatory elements in the promoter regions 2,000 bp upstream of the L. cubeba. were predicted by PlantTDFB software. [file DataSheet_1.zip › files/Figure s1.tiff]
